# Proteomic Study Between Interstitial Channels Along Meridians and Adjacent Areas in Mini-Pigs

**DOI:** 10.3390/biom15060804

**Published:** 2025-06-01

**Authors:** Feng Xiong, Shuyong Jia, Guangjun Wang, Shuyou Wang, Li Zhou, Qi Liu, Yaohua Shen, Na Tu, Shuxiu Zhu, Xiaojing Song, Weibo Zhang

**Affiliations:** 1 Institute of Acupuncture and Moxibustion, Jianghan University, Wuhan 430056, China; xiongfeng@jhun.edu.cn (F.X.); zhusx@jhun.edu.cn (S.Z.); 2 School of Medicine, Jianghan University, Wuhan 430056, China; 3 Institute of Acupuncture and Moxibustion, China Academy of Chinese Medical Sciences, Beijing 100700, China; 4 College of Acupuncture and Massage, Tianjin University of Traditional Chinese Medicine, Tianjin 301617, China; 5 College of Acupuncture and Massage, Beijing University of Traditional Chinese Medicine, Beijing 100029, China

**Keywords:** interstitial channels, stomach meridian, differential proteomics, fibrous connective tissues, mini-pigs

## Abstract

Objective: This study explores the material basis and biological functions of meridian interstitial channels in mini-pigs proximal to the stomach meridian by analyzing differential proteomics between interstitial channels and adjacent non-interstitial channel tissues. Methods: Liquid chromatography–mass spectrometry (LC-MS) under data-dependent acquisition mode was employed to analyze and identify the proteome of subcutaneous connective tissues along the stomach meridian and adjacent tissues. SWATH MS^ALL^ method and omicsbean online analysis platforms were used for protein quantification and differential proteomic analysis. Differential proteins were subjected to Gene Ontology annotation and KEGG pathway analysis to understand their functions and biological processes. Combining traditional Chinese meridian theory with modern meridian research, proteins most relevant to meridian functions were selected, and their expression levels were assessed using Western blotting. Results: GO annotation and KEGG pathway analysis revealed differences in molecular functions, biological processes, and metabolic pathways among differential proteins. Most downregulated proteins were enzyme functional proteins involved in amino acid metabolism (GOT1), adenosine nucleotide balance conversion (AK1), and calcium ion-binding processes (ANXA6). Most upregulated proteins were structural proteins in the extracellular matrix—collagen proteins (COL3A1, COL6A1, COL6A3, COL6A6, COL12A1, COL14A1) and proteoglycans (DCN, BGN, FMOD)—involved in influencing and regulating collagen fiber generation and arrangement. Intriguingly, almost all differential proteins were associated with gastrointestinal diseases, implying a pathological correlation of differential proteins in the stomach meridian interstitial channel. Conclusions: The stomach meridian interstitial channels in mini-pigs show 72 differentially expressed proteins compared to adjacent tissues. These differences include the upregulation of structural proteins and downregulation of functional proteins, potentially forming the molecular biological basis for the structural and functional specificity of meridians.

## 1. Introduction

Meridians are an important concept in traditional Chinese medicine (TCM). They are considered special physiological structures that are believed to serve as pathways for the transmission of Qi (according to traditional Chinese medicine, the nutrient-rich subtle substances flowing within the body can be regarded as the free liquid phase of interstitial fluid), blood, nutrients, and information, closely related to the physiological and pathological processes of the human body. The intricate biological mechanisms within meridians have been a focal point of research. Presently, studies on meridians primarily focus on visualization [1,2,3,4,5,6,7,8,9,10], biophysics [11,12,13,14], anatomy [15,16,17], and electrophysiology [18,19], and are still in the nascent stages of investigating protein specificity within meridian tissues. Zhou, L.H. [20] suggests that classical meridian pathways contain a systematically arranged, interrelated set of proteins. Feng, S.C. [21] discovered fibrous proteins at acupoints that participate in the generation and transmission of acupuncture effects, proposing that these fibrous proteins are integral components of the substance of meridians. Meng et al. [22] conducted a systematic study on the 29KD protein specific to sensory nerves, suggesting its association with many meridian functions. Studies have found common differential protein bands along the adipose tissue belts running along the conception vessel meridian (CV) and stomach meridian (ST), absent in adjacent non-meridian regions [23]. Additionally, research has shown significantly higher expression of gap junction protein Cx43 in the meridian area compared to adjacent non-meridian regions in rats [24]. These findings collectively suggest the existence of characteristic proteins within meridian tissues, possibly constituting a material foundation of the meridians.

However, the aforementioned studies have been limited to individual proteins when it comes to investigating characteristic proteins of meridians. To delve deeper into the study of meridian-specific proteins, we can introduce novel techniques. Differential proteomics is a high-throughput analytical method based on mass spectrometry technology used to study differences in protein expression levels among different samples. It possesses the advantages of comprehensive molecular profiling, functional pathway integration, and dynamic quantification precision. This technique has found wide application in the biomedical field. Proteomic studies of meridians represent a nascent research area. Pioneering work in this field has primarily focused on profiling differential protein expression in animal models of various diseases (e.g., inflammatory bowel disease, neuropathic pain) following acupuncture at meridian points [25,26,27,28,29,30]. They discovered specific protein differential expression acupuncture, which regulates relevant pathways to treat diseases. These studies primarily focus on pathological conditions. However, we observed differential proteomics between the normal physiological state of the CV in mice and adjacent non-meridian regions, finding upregulated protein expression in the meridians even under normal physiological conditions. These proteins potentially correlate with the biological functions of meridians [31]. All these findings indicate differential protein expression in meridians under both physiological and pathological conditions. Therefore, leveraging differential proteomic techniques could aid in identifying characteristic proteins associated with meridian functionality, contributing to a better understanding of the essence of meridians.

For objective research into the objective existence and essence of meridians, researchers such as Zhu et al. [32] and Mu et al. [33] separately discovered low-impedance points (LIP) that closely corresponded to the meridians in humans and mini-pigs. Meng et al. [34] traced meridians using Na^99m^TcO4 (^99m^TC) and found that the migration route of ^99m^TC closely aligned with classical meridian pathways. Zhang, W.B. [35], to explain the phenomenon of isotopic migration along meridians, speculated that meridians possess low hydraulic characteristics, allowing the convergence of tissue fluid towards the meridians and flow along them, based on Darcy’s law of fluid flow and the continuity equation. Subsequently, Zhang et al. [36,37], using biomechanical methods, demonstrated the low hydraulic characteristics of meridians. Combined with isotope tracing and alcian blue (AB) tracing techniques, they discovered low hydraulic interstitial channels along meridians in humans and mini-pigs [38]. A series of studies investigated the function of these channels in transporting fluids, nutrients, information, and drugs [39]. They also simulated the low electrical impedance and high conductive sound properties of meridians in a gel saline model [40]. In recent years, our team discovered meridian-like migration trajectories using AB dye in transparent fish [1,2]. Subsequently, using fluorescein sodium (FS) and fluorescence photography, we discovered migration along meridian trajectories after injection at LIPs along the CV in rats, mini-pigs, and humans [3,4,5,6,10]. Combining in vivo confocal microscopy techniques with conventional morphological methods, we observed that the structural basis for dye migration lay within fibrous connective tissues [41,42]. Building upon this foundation, this study presents the first analysis of differential protein expression in the subcutaneous connective tissue between the ST interstitial channels and adjacent non-meridian regions in mini-pigs, aiming to explore the material foundation and biological functionality of meridians.

## 2. Methods

### 2.1. Animals

Seventeen male Bama pigs weighing 11 ± 2 kg each, were provided by the Beijing Liulihe Kexing Experimental Animal Breeding Center (License for Experimental Animal Use: SCXK (Beijing) 2017-0003). Bama mini-pigs were chosen for their well-documented anatomical and physiological similarities to humans in skin thickness, subcutaneous connective tissue organization, and meridian distribution—advantages unattainable in rodent models due to anatomical disparities [38,42]. They were fed concentrated feed daily (Hope Feed Co., Ltd., Beijing, China)—150 g at 8:00 and 100 g at 20:00—with unrestricted access to water, and housed individually. The animal room was maintained at a temperature of 20 ± 2 °C and a humidity level of 50% ± 10% Rh. For the experiment, the mini-pigs were pre-administered a sellarazine hydrochloride injection (Huamu Animal Health Care Co., Ltd., Changchun, China) for light anesthesia, and continuous anesthesia was maintained using a mixture of isoflurane (Yipin Pharmaceutical Co., Ltd., Shijiazhuang, China) and oxygen inhalation. The entire experiment was approved by the Ethics Committee of the Institute of Acupuncture and Moxibustion, China Academy of Chinese Medical Sciences (Ethics Approval Number: D2022-04-14). All experiments were conducted following guidelines for the care and use of experimental animals.

### 2.2. Tracer Injection and Observation

After fully anesthetizing three mini-pigs, they were positioned laterally on the experimental table. Following shaving, the low-impedance lines of ST in the mini-pigs were measured using the WQ6F30 meridian locator (Donghua Electronics Factory, Beijing, China). Subcutaneous injection of 1% AB, 1 mL (1 mL/3 h) (Sigma, Livonia, MI, USA) was administered at the LIPs. After 3 h, the skin was incised with a surgical blade to collect images.

### 2.3. In Vivo Confocal Observation of Micro-Migration Trajectory Structure

Four mini-pigs were injected with 100 μL of 1% FS (Guangxi Wuzhou Pharmaceutical Co., Ltd., Wuzhou, China; product batch number: 181102) at the LIP of ST. At 20 min post-injection, a small cross (approximately 2 mm in depth) was incised near the injection site, exposing the subcutaneous fascial layer containing the fluorescent dye. The scanning probe was positioned in the fluorescent area, adjusting parameters such as scan depth, white balance, and laser power to obtain the clearest image. Observation of 2–3 points was made along each migration line. FS was injected into adjacent non-meridian regions, and images were captured using the same method. The tissue structure images of fluorescent migration lines and adjacent areas were analyzed using the in vivo laser confocal imaging system (ViewnVivoB30, OptiScan, Mulgrave, Australia). Operator 1 performed a second imaging observation five minutes after the initial examination, while operator 2 followed the same procedure, disregarding the results from the first operator. The imaging results obtained by operator 1 were used for image analysis.

### 2.4. Fresh Frozen Sections of Migration Trajectories and MASSON, EVG Staining Observation

Tissue samples (1 × 1.5 × 1 cm) were collected from the FS migration lines and adjacent areas without migration lines in seven mini-pigs. A portion of the samples were rapidly frozen using a cryostate (Cryotome FSE, Thermo Fisher, Waltham, MA, USA). After embedding in optimal cutting temperature (OCT, Richard-Allan Scientific neg50, Thermo Fisher, Waltham, MA, USA) compound, tissue blocks were sectioned using a microtome. Sequential sections in sagittal and transverse planes were prepared with a thickness of 20 μm. After slide completion, they were air-dried in darkness, coverslipped, and observed and photographed under a fluorescence microscope (BX61VS 120-S6-W, Olympus, Tokyo, Japan). Another portion of each sample, fixed in 4% paraformaldehyde, was embedded in paraffin, and 6 μm thick continuous sections were produced, with 2 slides subjected to Masson’s staining and 2 slides subjected to EVG staining. Structural observations of migration lines, adjacent non-meridian areas, and the distribution and shape of collagen fibers were conducted under an optical microscope. All morphological inspections were performed by experimental technicians with extensive experience in tissue morphology analysis, without prior knowledge of the experimental purpose.

### 2.5. Extraction of Connective Tissue from the Interstitial Channels of the ST and Adjacent Non-Meridian Areas

**Figure 1:**
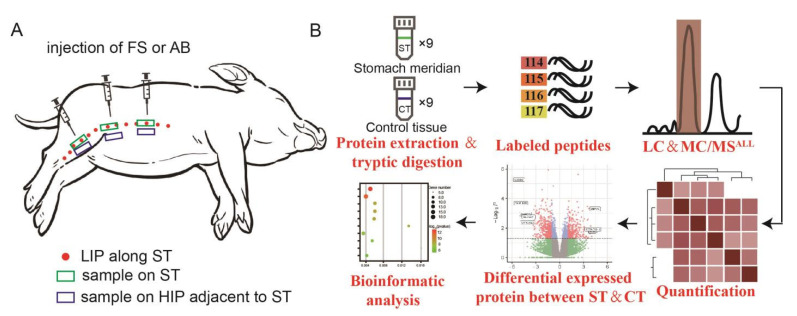
Overview diagram of experimental procedures. (**A**) Schematic diagram of the locations of the mini-pig samples. Red dots indicate the LIPs of stomach meridian, green boxes represent the locations of ST samples, and blue boxes indicate the adjacent non-meridian tissue locations (CT). (**B**) Proteomic strategy for the differential protein analysis of subcutaneous connective tissue in ST and CT. ST refers to the subcutaneous connective tissue of the stomach meridian, while CT represents the subcutaneous connective tissue adjacent to the non-meridian area.

After anesthetizing the three mini-pigs, we obtained tissue samples from the low-impedance line of ST and the adjacent non-meridian site of the ST (high impedance point, 5 cm away from the low-impedance line of ST) (Control tissue, CT). Each mini-pig provided 3 samples of both ST and CT, totaling 18 samples. The collected tissue had a width of approximately 5 mm and a thickness of about 2 mm, and was stored in a −80 °C freezer (Figure 1A).

### 2.6. Preparation, Extraction, and Quantification of Total Protein from Samples

Appropriate tissue blocks were taken, minced, and washed with phosphate-buffered saline (PBS) containing protease inhibitors (4 °C). Centrifugation (5810R Multi-functional Benchtop Centrifuge, Eppendorf, Hamburg, Germany) was carried out at 3000 rpm, 4 °C for 3 min, and the supernatant was discarded. This washing process was repeated once. In 1 mL of cold lysis buffer, 10 μL of phosphatase inhibitors, protease inhibitors, and phenylmethylsulfonyl fluoride (PMSF) were added, mixed well, and set aside on ice. Approximately 0.1 g of pretreated tissue fragments were added to 1.0 mL of lysis buffer, placed in an ice bath, and sonicated using an ultrasonic probe disruptor (600 w, sonication 5 s, pause 5 s, total duration 2 min). Post-sonication, the disrupted tissue mixture was placed in a shaker at 160 rpm in an ice bath for 30 min. The lysate obtained after disruption was centrifuged at 14,000 rpm, at 4 °C for 40 min, and the supernatant, constituting the total protein solution for both ST and CT samples, was collected. The extracted supernatant was stored in a −80 °C freezer for later use. The total protein concentration in the supernatant was quantified using the BCA assay method (BCA Protein Concentration Determination Kit, Solarbio, Beijing, China).

### 2.7. Protein Precipitation

Each sample was taken with 100 μg of protein, and an appropriate volume of 20% trichloroacetic acid (TCA) was added to achieve a final concentration of 10%. The mixture was thoroughly mixed and incubated at −20 °C for 2–3 h to allow protein precipitation. Subsequently, the sample was centrifuged at 14,000 rpm for 30 min at 4 °C, and the supernatant was discarded. The precipitate was suspended in 1 mL of acetone (4 °C), sonicated for 5 min, and incubated overnight at −80 °C. The sample was centrifuged again at 14,000 rpm for 30 min at 4 °C, the supernatant was removed, and acetone was evaporated at room temperature.

### 2.8. Protein Sample Preprocessing and Peptide Solution Preparation

Each sample underwent the following procedure for preprocessing and preparation of peptide solutions. A total of 70 µL of 0.2% Rapigest SF in 50 mM ammonium bicarbonate (ABC) solution was added to the protein sample and sonicated for dissolution. The remaining acetone was evaporated at 37 °C and 300 rpm. Then, 8 µL of 100 mM Tris (2-carboxyethyl) phosphine (TCEP) in 50 mM ABC solution and 2 µL of 50 mM ABC solution were added to achieve a final concentration of 10 mM. The mixture was vortexed and incubated at 60 °C for 30 min, cooled to room temperature, and then 9 µL of 100 mM iodoacetamide (IAA) in 50 mM ABC solution and 1 µL of 50 mM ABC solution were added to reach a final concentration of 10 mM. After thorough vortexing, it was incubated in a dark room at room temperature for 30 min. Then, 2.5 µL of 0.4 µg/µL restriction-grade trypsin (enzyme:protein = 1:100) (Worthington, OH, USA) was added and mixed uniformly before incubating at 37 °C for 2 h. Subsequently, another 2.5 µL of 0.4 µg/µL trypsin (enzyme:protein = 1:50) was added, mixed thoroughly, and left to incubate overnight at 37 °C. After digestion, 5 µL of 10% TCA solution was added, mixed, and the enzyme reaction was stopped at 37 °C and 140 rpm for 30 min. The supernatant containing the digested peptides was transferred to a 10 kDa ultrafiltration membrane, centrifuged at 14,000× *g* for 15 min, and the filtrate was transferred to a new reaction tube, yielding a sample solution with a concentration of 1 µg/µL (filtration recovery approximately 90%). The samples were aliquoted and stored at −80 °C.

### 2.9. Establishment of DDA Mass Spectrometry Data Acquisition Method for Mini-Pigs

A total of 10 µL of peptide solutions from ST and CT samples were mixed thoroughly. Using a nano-high-performance liquid chromatography (HPLC) system (Eksigent 400 nano HPLC) (Eksigent, AB Sciex, Dublin, CA, USA) coupled to a tandem time-of-flight mass spectrometer (5600 + TripleTOF) (AB Sciex, Dublin, CA, USA), data-dependent acquisition (DDA) mode was employed to optimize the chromatographic and mass spectrometric system parameters by collecting data on the mixed samples.

Initially, a precise volume of 10 µL of the mixed peptide solution was injected into the nano-HPLC enrichment column (350 µm × 0.5 mm, Chrom XP C18~3 µm, 120 A, Eksigent). The peptides were washed with a mobile phase consisting of formic acid–acetonitrile–water (0.1:2:98, *v*/*v*/*v*) for 10 min at a flow rate of 0.5 µL/min, aiming to enrich and desalt the mixed peptides. Subsequently, the flow path was switched, and the peptides underwent gradient separation using a capillary chromatography column (Beijing LP Science & Technology, Beijing, China, 75 µm × 20 cm, Sunchrom C18-5 µm, 120 A) at an elution rate of 0.3 µL/min.

Subsequently, in the high-resolution mode, primary spectra (MS1) were scanned and collected within the range of 250 to 1250 *m/z*. The parameters for time-of-flight mass spectrometry (TOF MS) were set as follows: nano electrospray, nebulizing gas (GS1) at 10, curtain gas (CUR) at 30, ion spray voltage (ISDF) at 2500 V, interface heat (IHF) at 150, declustering potential (DP) at 100 V, collision energy (CE) at 10 eV, accumulation time at 250 ms, mass tolerance (MT) at 50 mDa, dynamic exclusion set at 15 s for acquisition time, and charge state ranging from 2 to 5. In the high-sensitivity mode, fragment ions were collected in the second level spectra (MS2) within the mass-to-charge ratio (*m/z*) range of 100 to 1500, with a cycle time of 100 ms.

### 2.10. Protein Qualitative Identification

To enhance the quantity of protein identification, a method of online grouping after merging subgroup samples was adopted. Specifically, peptide solutions from ST and CT subgroups were combined as follows: ST1-ST3, ST4-ST6, ST7-ST9, CT1-CT3, CT4-CT6, and CT7-CT9. The range for collecting primary spectra in the DDA method was divided into 250 to 570 *m/z* and 565 to 1250 *m/z*. Mass spectrometry data were collected following the method mentioned above, acquiring a total of 12 online grouped mass spectrometry datasets. Alongside the mixed sample results mentioned above, the ProteinPilot 5.0.2 software was used to search for these 13 datasets against a protein library downloaded from the National Center for Biotechnology Information (NCBI), specifically the Sus scrofa (pig) protein database (.fasta), for protein identification (16 October 2022). The parameters were set as follows: sample type: identification; cysteine acetylation modification: iodoacetamide; enzyme digestion: restriction enzyme; instrument model: TripleTOF5600; special parameters: none; species: none; search type: ID; protein identification result quality control threshold [unused protscope (conf)]: 0.05 (10%); simultaneous false discovery rate (FDR) analysis was conducted. The search results formed the identified protein library.

### 2.11. Sample Protein Quantitative Data Collection

The SWATH (Sequential Window Acquisition of all Theoretical fragment ions) label-free method was employed for the quantitative assessment of the identified proteins. Based on the qualitative data from mixed samples under Section 2.10, the SWATH method was established. In DDA mode, a data collection of mixed peptides within the range of 250–1250 *m/z* was conducted. Post-processing with PeakView 2.0 (AB Sciex) was employed to handle sub-ion *m/z* and signal intensities. Subsequently, the Variable Window Calculator (V 0.2 112513) (AB Sciex) computed the size of the SWATH-MS dynamic acquisition windows. The parameters were set as follows: 60 windows, minimum *m/z* 400, maximum *m/z* 1250, window overlap of 0.5 Da, and collision energy spread (CES) at 15 V. The series of data collection windows were thus calculated. Under high-sensitivity mode, MS2 spectra were collected within the 100–1500 *m/z* range, with parameters set as follows: a cycle time of 1.42 s, CES at 15 V, charge at 2, and CE at 10 V. Finally, the nano-LC and SWATH MS ^ALL^ methods were employed to conduct SWATH data collection on the 18 samples of ST and CT peptides, with each sample undergoing SWATH data collection thrice for replicability.

### 2.12. Data Processing and Bioinformatics Analysis

Utilizing the Peak View SWATH Processing Micro App (AB Sciex, v.1.0.0.1409), the SWATH data collected from ST and CT samples underwent segmented processing, correcting retention times. For extracting proteins quantifiable by SWATH, along with their corresponding peptide sequences and fragment ion details, from the identified protein library, SWATH data processing parameters were set as follows: a minimum of 6 quantifiable peptides per protein, each peptide having at least 6 quantifiable fragment ions, a 15-min extraction window, a mass-to-charge ratio deviation of 50 mDa, a confidence interval of 95%, and a 1.0% FDR.

Using the omicsbean online analysis platform, relative quantitative comparisons were made between ST and CT protein quantification data lists. Initial normalization of all quantitative protein peak areas was conducted, followed by principal component analysis (PCA) based on groupings, combining Clustering and HeatMap functions for experiment data quality control. Subsequently, a *t*-test was employed, treating the CT group as B and the ST group as A, using the B:A ratio (fold change, FC) and *p*-value as criteria for screening differentially expressed proteins. Proteins with *p* ≤ 0.05 and FC ≥ 1.5 or ≤ 0.67 were selected, ultimately identifying differentially expressed proteins in the ST and CT groups.

After converting the NCBI protein identifiers of the screened differential proteins to UniProt numbers, the omicsbean online data analysis platform was used for bioinformatics analysis of identified proteins. This analysis included Gene Ontology (GO) annotations, Kyoto Encyclopedia of Genes and Genomes (KEGG) pathway analysis, and Protein–Protein Interaction (PPI) analysis. Referencing the UniProt database, an analysis was conducted on the functional aspects and biological processes involving the differentially expressed proteins.

Drawing from the morphological structure of the low-impedance line tissue along the ST obtained in the experiment, TCM theories regarding meridian principles and ST characteristics, contemporary research on meridian substance studies, and the functions and biological processes involving differential proteins, an analysis was performed to investigate the correlation between these differential proteins and meridian function, exploring the protein-based biological foundation of meridians.

### 2.13. Statistical Analyses

All statistical analyses were conducted using SPSS 22.0 and GraphPad Prism 9. Differential protein expression between ST and CT groups was determined via unpaired two-tailed Student’s *t*-tests, with Benjamini–Hochberg correction (FDR < 0.05) and a fold change threshold of |log_2_(FC)| ≥ 0.58 (|FC| ≥ 1.5). Multivariate analyses included PCA and hierarchical clustering (Euclidean distance, Ward’s linkage) to assess group separation. Pathway enrichment (GO, KEGG) employed Fisher’s exact test (−log10(P) > 1.3. Technical reproducibility was ensured by triplicate SWATH-MS runs (CV < 15%) and TIC normalization.

## 3. Results

### 3.1. Migration Characteristics and Morphological Observation After Dye Injection into Lips Along the ST in Mini-Pigs

**Figure 2:**
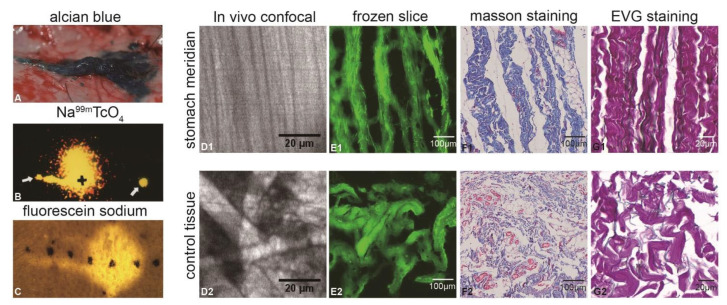
Images of the mini-pig’s LIP after dye injection and microstructural images. (**A**) Image of dye migration in the subcutaneous connective tissue layer after ST’s LIP injection of AB. (**B**) γ-camera captured image after ST’s LIP injection of 99 mTc pertechnetate “Reproduced from [38], with the permission of JAMS”. (**C**) Image of migration after ST’s LIP injection of FS “Reproduced from [4], with the permission of SCP”. (**D1**,**D2**) In vivo confocal images at the ST and adjacent non-meridian (control tissue, CT) locations, respectively. (**E1**,**E2**) Frozen fluorescent staining histological slice image at the ST and CT, respectively. (**F1**,**F2**) Masson’s trichrome histological slice images respectively at the ST and CT. (**G1**,**G2**) EVG staining histological slice image at the ST and CT, respectively.

Three hours after injecting AB into the LIPs along the ST in mini-pigs, upon lifting the skin, we observed a linear migration of AB roughly following the path of the low-impedance line (Figure 2A), extending to approximately 4.5 cm. This migration pattern was consistent with previous observations following injections of 99 mTC (Figure 2B) and FS (Figure 2C) into the LIPs along the ST. Via in vivo confocal microscopy examination of both the ST low-impedance line area and the adjacent non-meridian region, the fluorescent dye was found to be situated above the fibrous connective tissues. However, the fibers in the low-impedance region of the ST displayed a parallel arrangement (Figure 2(D1)), while those in the non-meridian region appeared comparatively more intricate (Figure 2(D2)). Observations of frozen fluorescent-stained slices of the ST (Figure 2(E1)) and paraffin sections stained with Masson’s trichrome (Figure 2(F1)) and EVG (Figure 2(G1)) were consistent with the in vivo observations. In all cases, a substantial presence of parallelly distributed collagen fibers and elastic fiber tissues was observed, suggesting that these tissues might serve as the material basis for dye migration.

### 3.2. Identification of Protein Composition in the ST and CT

Peptide identification was performed with a false discovery rate (FDR) threshold of 1% at the peptide level, resulting in 12,781 high-confidence peptides. Protein inference was subsequently applied, maintaining a 1% FDR at the protein level, which identified 1073 protein groups. After rigorous filtering to remove reverse database matches (REVERSED) and contaminants, a final dataset of 1067 proteins was retained for downstream analysis.

### 3.3. Analysis of Differential Proteins Between the ST and CT

Protein quantification was performed using label-free SWATH-MS (data-independent acquisition). Tissues along the ST and adjacent CT were sampled from nine biological replicates per group. Each biological replicate was analyzed in triplicate technical runs, generating 54 SWATH datasets (9 ST × 3 runs + 9 CT × 3 runs). Chromatographic consistency between the DDA spectral library and SWATH runs was validated by high retention time correlation (median R^2^ = 0.98 across all peptides). Of the 1067 proteins identified in the DDA library, 876 (82.1%) were reliably quantified (CV < 20% across technical replicates), while 191 proteins were excluded due to missing values in >50% samples.

**Figure 3:**
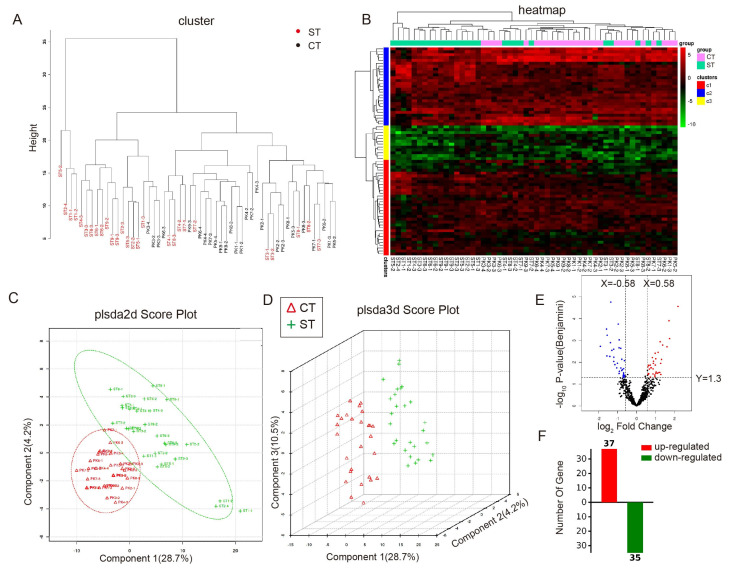
Image processing of differential proteomics data. (**A**) Cluster analysis cluster diagram. Red represents the ST group, while black represents the CT group. (**B**) Cluster analysis heatmap. Cyan represents the ST group; pink represents the CT group. Red and black sections indicate upregulated proteins, while green indicates downregulated proteins. (**C**,**D**) PCA analysis plsda 2d scatter plot and 3d scatter plot. Red represents the CT group, and green represents the ST group. (**E**,**F**) Volcano plot and statistical graph displaying differential protein expression. Blue and green indicate downregulated expression, while red indicates upregulated expression.

The relative quantitative comparison between ST and CT groups was performed using the OmicsBean online (chttp://www.omicsbean.cn, 12 November 2022) analysis platform. Initially, normalization analysis was applied to all quantified protein peak areas. Cluster analysis through the cluster diagram (Figure 3A) and heatmap (Figure 3B) revealed a certain degree of clustering between the two groups but generally belonged to distinct categories. Principal Component Analysis (PCA) in the plsda2d scatter plot (Figure 3C) showed evident separation between ST and CT groups with minimal overlap (Q^2^ = 0.72, R^2^Y = 0.89; permutation test *p* < 0.01). The results from the plsda3d scatter plot indicated that ST and CT groups belonged to two different categories (Figure 3D). After T-test analysis, 72 proteins exhibited differential expression between ST and CT, with 37 upregulated and 35 downregulated proteins (Figure 3E,F).

### 3.4. Differential Protein GO Function Enrichment Analysis and KEGG Pathway Analysis

**Figure 4:**
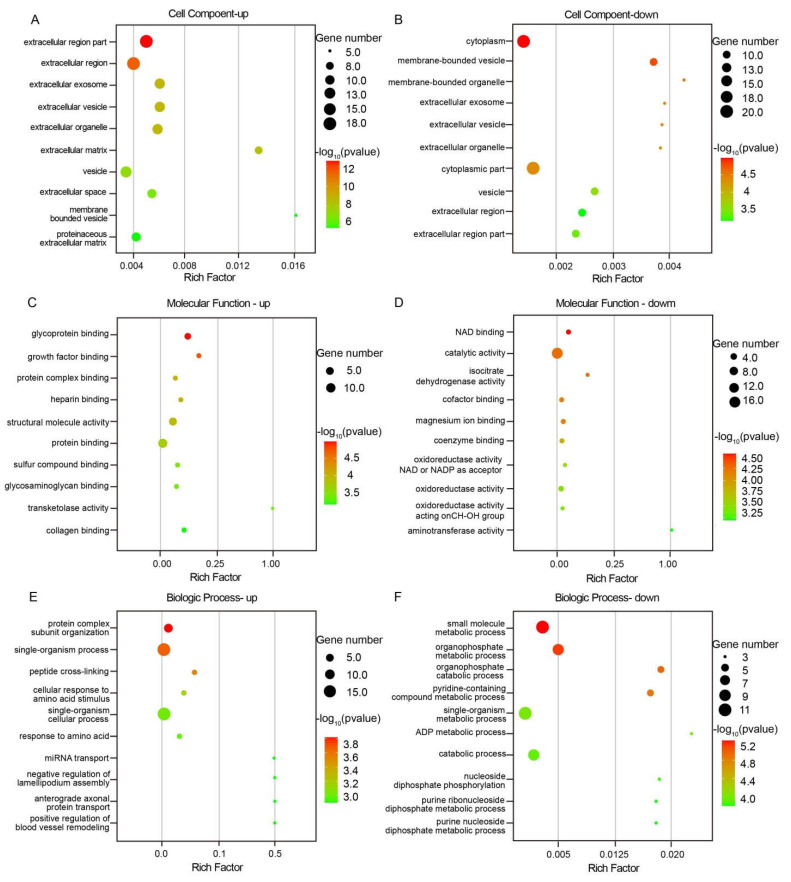
GO enrichment analysis of significantly different proteins between ST and CT tissues. (**A**,**B**) Bubble charts illustrating the CC aspects of upregulated and downregulated proteins, respectively. (**C**,**D**) Bubble charts representing the MF aspects of upregulated and downregulated proteins. (**E**,**F**) Bubble charts displaying the BP aspects of upregulated and downregulated proteins.

The 72 differentially expressed proteins with known functions obtained from the comparison between ST and CT groups underwent GO annotation. This initial analysis aimed to elucidate the molecular functions (MF), cellular components (CC), and biological processes (BP) these differentially expressed proteins might be involved in. The GO analysis results revealed that the upregulated differential proteins in mini-pigs’ ST and CT samples were primarily distributed in the cellular components associated with the extracellular environment, such as the extracellular region part, extracellular region, extracellular exosome, extracellular vesicle, extracellular organelle, and extracellular matrix (Figure 4A). In contrast, the downregulated differential proteins were predominantly located in intracellular compartments, including the cytoplasm, membrane-bounded vesicle, membrane-bounded organelle, and cytoplasmic part (Figure 4B).

The molecular biological functions enriched in upregulated differential proteins primarily involved protein binding aspects, such as glycoprotein binding, platelet-derived growth factor binding, protein complex binding, heparin binding, sulfur compound binding, glycosaminoglycan binding, and collagen binding (Figure 4C). On the other hand, the downregulated differential proteins were mainly enriched in molecular functions like NAD binding, catalytic activity, isocitrate dehydrogenase activity, cofactor binding, magnesium ion binding, coenzyme binding, oxidoreductase activity acting on the CH-OH group of donors, NAD or NADP as acceptor, oxidoreductase activity, oxidoreductase activity acting on CH-OH group of donors, and transaminase activity (Figure 4D).

The biological processes enriched in upregulated differential proteins involved protein complex subunit organization, single-organism process, peptide cross-linking, cellular response to amino acid stimulus, single-organism cellular process, response to amino acid, miRNA transport, negative regulation of lamellipodium assembly, anterograde axonal protein transport, and positive regulation of blood vessel remodeling (Figure 4E). Conversely, the biological processes enriched in downregulated differential proteins included small molecule biosynthetic process, organophosphate metabolic process, organophosphate catabolic process, pyridine-containing compound metabolic process, single-organism metabolic process, ADP metabolic process, catabolic process, nucleoside diphosphate phosphorylation, purine ribonucleoside diphosphate metabolic process, and purine nucleoside diphosphate metabolic process (Figure 4F). Figure 3 displays the top 10 enriched components in CC, MF, and BP based on −lg*p* ranking.

**Figure 5:**
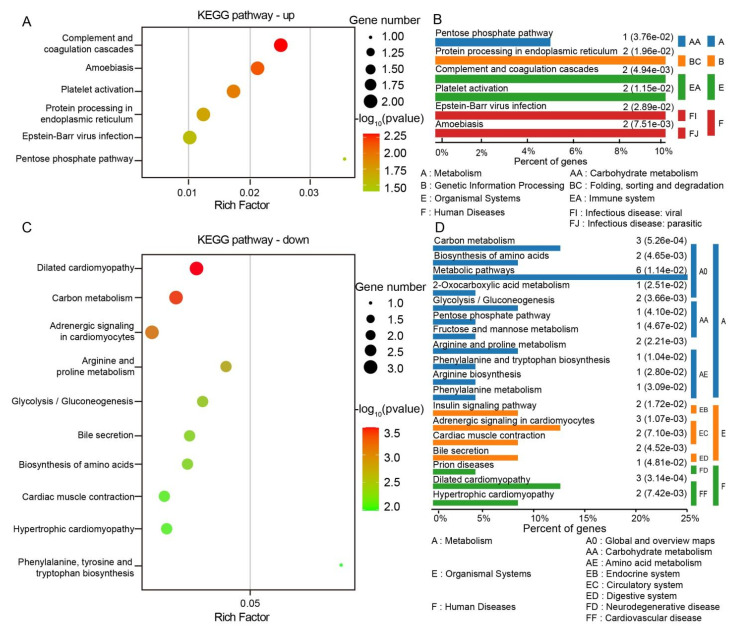
Illustrates the KEGG metabolic enrichment analysis of significantly different proteins between ST and CT tissues. (**A**,**B**) Bubble chart and hierarchical enrichment plot, respectively, for upregulated proteins. (**C**,**D**) Bubble chart and hierarchical enrichment plot, respectively, for downregulated proteins.

After conducting KEGG analysis on the significantly different proteins between ST and CT tissues, it was found that the signaling pathways enriched in upregulated proteins mainly involved the complement and coagulation cascades, amoebiasis, platelet activation, protein processing in the endoplasmic reticulum, Epstein–Barr virus infection, and the pentose phosphate pathway. On the other hand, the pathways enriched in downregulated proteins were primarily associated with dilated cardiomyopathy, carbon metabolism, adrenergic signaling in cardiomyocytes, arginine and proline metabolism, glycolysis/gluconeogenesis, bile secretion, biosynthesis of amino acids, cardiac muscle contraction, hypertrophic cardiomyopathy, and phenylalanine, tyrosine, and tryptophan biosynthesis. Figure 5 displays the top 10 enriched components in KEGG pathway analysis based on −lg*p* ranking (only 6 pathways had a *p*-value less than 0.05 in the upregulated KEGG signaling pathways).

Combining TCM’s description of the physiological functions of the ST, it is acknowledged that the ST travels extensively, connecting with numerous visceral meridians. It is considered the source of the body’s Qi and Blood, and hence associated with strong pulses and abundant blood flow, indicative of vigorous Qi and abundant Yang (representing vigorous energy metabolism). When the Blood is abundant, it nourishes the tendons and vessels, supporting the robust development of muscles and bones (governs the nourishment of muscles and bones) and parallel fibers in the dye migration pathway (an essential component of the extracellular matrix). Based on GO annotations and KEGG pathway results, proteins possibly related to the structure and function of the stomach meridian were selected. Among the differentially expressed proteins, those involved in amino acid synthesis, metabolic processes, purine ribonucleotide metabolic processes, calcium ion binding-related functions, and structural proteins contributing to the composition of the extracellular matrix are preliminarily identified as potentially influential in the structural and functional specificity of the meridian.

## 4. Discussion

### 4.1. Structural-Functional Dichotomy of Meridian Tissues: ECM Signaling and Intracellular Metabolic Reprogramming

Through high-resolution protein identification and data-independent quantitative methods, this experiment systematically investigated differential proteomics between meridian and non-meridian tissues in mini-pigs. The findings indicate that upregulated differentially expressed proteins are mainly distributed in the extracellular environment, such as the ECM. Research suggests that the extracellular matrix does more than previously thought, functioning not just as inert support but containing numerous signaling molecules actively involved in controlling cell growth, polarity, shape, migration, and metabolic activities. Certain matrix components also impact vascular generation and immune regulation [43,44,45,46]. Conversely, downregulated proteins are predominantly enriched within the cell. These cellular structures participate in the transport, recognition, and transmission of substances such as neurotransmitters, cytokines, and ions. The molecular function of upregulated proteins primarily involves protein binding, whereas downregulated proteins are mostly associated with enzymatic activity. Upregulated proteins participate in biological processes such as protein synthesis, while downregulated proteins are involved in various substance metabolic processes.

### 4.2. Metabolic Adaptation and Redox Signaling in Meridian Tissues: A Nexus of Energy Homeostasis, Oxidative Stress, and Calcium Dynamics Underlying Acupuncture Efficacy

This study focuses on the interstitial channels of meridians, where one of the material bases is the extracellular matrix. Meridians are channels in the body that facilitate the circulation of Qi and blood, connecting the internal organs with the body surface and various parts of the body, constituting the body’s regulatory system. All substance transport and information transmission within a biological organism require energy consumption. Modern research results indicate that tissues distributed along the superficial meridian pathways exhibit distinctive biological characteristics compared to non-meridian areas. These include enhanced infrared radiation at higher body surface temperatures [47], increased ultraweak photon emissions, elevated oxygen consumption [48], heightened carbon dioxide release [49], and a richer microcirculation [50]. These various characteristic biophysical phenomena observed in meridian tissues suggest highly active energy metabolism within these regions. Moreover, tissues distributed along the meridians demonstrate high nerve ending density, elevated concentrations of K^+^, Na^+^, Ca^2+^ ions, and aggregation of mast cells [51]. These factors provide a material foundation for the active cellular metabolism in tissues along the meridians. In the active metabolic processes of tissues, there is inevitably a substantial involvement of ATP.

ATP is an important high-energy phosphate compound, which provides energy for various life activities [52,53,54]. Representatively, the downregulated expression enzymes such as Aspartate Aminotransferase 1 (GOT1) and Isoenzyme 1 (AK1) are related to ATP production. GOT1 participates in various amino acid metabolic pathways, playing a significant role in maintaining cellular redox balance and regulating amino acid metabolism [55,56]. The downregulation of such enzyme systems, coupled with the upregulation of downstream metabolic product-related proteins (histidine-rich glycoprotein, HRG, Log_2_Fold change = −0.698; proline and arginine-rich end leucine-rich repeat protein, PRELP, Log_2_Fold change = −0.748), suggests excessive consumption due to active amino acid metabolism in the tissues along the meridians of mini-pigs. Active amino acid metabolism aids in providing energy for life activities through oxidative pathways and in metabolically clearing certain substances. Additionally, GOT1 helps reduce the concentration of glutamate salts in the blood and brain, safeguarding the brain’s nervous system [57], and modulates cysteine and alpha-ketobutyric acid levels [58,59].

AK1 is an intracellular phosphotransferase that regulates the ratio of different purine nucleotides [60,61]. Through phosphate transfer, it modulates multiple energy-dependent or nucleotide-signaling-related behaviors both inside and outside cells, playing a crucial role in the homeostasis of purine nucleotide metabolism. The downregulation of such enzyme systems, coupled with the mentioned active amino acid metabolism, suggests abundant ATP content in the organism, leading to decreased enzyme activity and weakened oxidative phosphorylation. Elevated ATP levels promote biological processes such as cellular activities (DNA transcription, translation), signal transduction, and transmembrane substance transport, contributing to the robust activity of the ST, the integration of bodily functions, and the high-energy metabolism characteristic of the meridian tissues [62,63].

However, in cases of emergency hypoxia or intense physical activity, energy is primarily provided by glycolysis [64,65]. During acupuncture stimulation, the spasm and contraction of acupoint tissues are in a stressed state, requiring local tissues to rely mainly on glycolysis to meet metabolic energy demands. Additionally, highly metabolically active cells such as neurons and glial cells require energy from glycolysis, even in the absence of oxygen [66,67,68]. This experiment also observed differential protein expression involved in the glycolysis/gluconeogenesis metabolic pathways in meridian tissues, exemplified by fructose 1,6-biophosphatase 2 (FBP2). FBP2 regulates the synthesis of glucose/glycogen precursors [69,70,71]. Studies have indicated that the absence of FBP2 in soft tissue sarcoma cells can increase glucose absorption and utilization, suggesting FBP2’s role in inhibiting glycolysis [72,73]. Reduced FBP2 enzyme activity could alleviate the inhibition of glycolysis, leading to an increase in intracellular glycolytic activity [74]. In summary, the downregulation of FBP2 in the ST tissues of mini-pigs suggests a potentially more significant glycolytic metabolism in these tissues compared to non-meridian tissues. This could ensure normal metabolic activity and information transmission of cells in meridian tissues, contributing significantly to the manifestation of meridian phenomena and acupuncture effects.

During the process of oxidative phosphorylation, where a substantial amount of ATP is synthesized, electrons leaked from the mitochondrial electron transport chain combine with O_2_ to produce superoxide anions [75]. Excessive generation of these anions can lead to tissue damage. This experiment observed an upregulation of superoxide dismutase (SOD), which catalyzes the dismutation of O_2−_ into H_2_O_2_ and O_2_, thereby protecting cells from damage caused by superoxide anions [76,77]. Reactive oxygen species (ROS) generated also need to be eliminated by peroxidases, reducing them to H2O and O_2_. However, intriguingly, this experiment observed the downregulation of peroxiredoxin 6 and glutathione peroxidase 1, indicating a higher content of superoxide radicals in meridian tissues. Guo et al. [9,78] marked the presence of high concentrations of superoxide radicals in the meridian tissues of the rat abdominal wall using a superoxide radical probe, suggesting active oxidative stress reactions in these areas. Yet, how the cells in meridian tissues tolerate such active oxidative stress reactions requires further exploration. Many researchers [79,80,81] believe that acupuncture exhibits antioxidant properties in the human body, but the mechanisms of these antioxidant effects on meridians and their role remain to be elucidated, providing potential avenues for further investigation.

Ca^2+^ serve as crucial intracellular secondary messengers, participating in almost all biological processes within an organism [82,83,84,85]. This study observed an upregulation of ANXA6, a calcium-binding protein known to associate with plasma and intracellular membranes, participating in cellular cytoskeleton movement, enzyme regulation, intracellular signal transduction, regulation of cell growth and proliferation, and the formation of atypical Ca^2+^ channels, among other crucial physiological activities. Research has linked the biological activity of Ca^2+^ in acupoint tissues to meridian activity and acupuncture effects [86,87]. Studies by Guo et al. and others observed higher Ca^2+^ concentrations in rabbit acupoints compared to non-acupoints, with significantly elevated Ca^2+^ levels in acupoints after acupuncture compared to non-acupoints. Moreover, there was a trend of Ca^2+^ redistribution along the meridians after acupuncture [88]. Lowering Ca^2+^ concentrations by chelating Ca^2+^ along acupuncture points or related meridians inhibited mast cell recruitment and degranulation in acupuncture areas, leading to the loss of acupuncture’s effects on visceral regulation [89]. Furthermore, blocking voltage-gated Ca^2+^ channels on cell membranes or inhibiting calmodulin’s activity in acupoint tissues can influence the effects of acupuncture [90]. Numerous studies have established that higher Ca^2+^ levels in acupoint areas are critical chemical factors in the generation of meridian phenomena and acupuncture effects. The biological functions of Ca^2+^ occur through a series of cascade reactions initiated by transmembrane Ca^2+^ transport within cells [91]. The alteration of intracellular and extracellular Ca^2+^ concentrations is a crucial factor influencing Ca^2+^ transmembrane transport, and the biological activity of calcium channel proteins and calcium-binding proteins on cell membranes significantly impacts these fluctuations in Ca^2+^ concentrations [92,93]. These findings suggest the active involvement of calcium-binding proteins in the biological process of Ca^2+^ transmembrane transport in meridian tissues, providing a biological basis for the occurrence of meridian phenomena and acupuncture effects.

### 4.3. Structural Blueprint of Meridian Channels: Collagen–Proteoglycan Networks as Molecular Scaffolds Linking ECM Architecture to Gastrointestinal Pathobiology

Besides the aforementioned differential expression of functional proteins within cells, a category of structural proteins also exhibits differences, such as collagen and proteoglycans found in the extracellular matrix (ECM). The ECM comprises large molecular substances secreted by cells into the extracellular interstitium, forming a complex network that supports and connects tissue structures and regulates tissue development and cell physiology. The ECM consists of proteins and proteoglycans/glycosaminoglycans in human cells that provide structural support [94,95]. Collagen, the most abundant family member in the ECM, is extremely diverse in structure and function, with 28 known subtypes (collagen I to XXVIII) assembled from 46 different gene products [96,97,98,99,100]. This experiment observed upregulated expression of collagen type III (COL3A1), collagen type VI (COL6A1, COL6A3, COL6A6), collagen type XII (COL12A1), and collagen type XIV (COL14A1) in the gastric meridian connective tissue. Type III collagen belongs to fibrillar collagen, type VI collagen belongs to beaded filament collagen, and types XII and XIV collagen belong to fibril-associated collagens.

In the ECM, type III collagen monomers form large molecular fibers, which aggregate to form fibers, creating a robust supportive structure [101,102]. Type VI collagen is a special kind of beaded filament collagen [103,104], creates a network of beaded microfibrils interacting with other ECM molecules, providing delicate structural support [105]. Type VI collagen acts not only as a structural protein but also as a signaling protein. Mutations in the type VI collagen gene are associated with muscular weakness disorders; for instance, mutations in COL6A1 can lead to Bethlem myopathy [106] and Ullrich congenital muscular dystrophy [107]. Type XII collagen is believed to alter the interaction between type I collagen fibrils and the surrounding matrix [108,,109,110]. In this sense, it is highly expressed in mechanically functional tissues, where it is thought to act as a regulator of biomechanical properties [111]. Recently, it was found that mutations in COL12A1 lead to alterations in connective tissue, presenting phenotypically similar to type VI collagen-related myopathies [112], including excessive joint laxity, mild muscle weakness, and joint contractures [113]. Type XIV collagen is a large molecular glycoprotein associated with mature collagen fibers, and the COL14A1 gene is closely related to core proteoglycans, playing a role in the development of connective tissues.

Decorin (DCN), lumican (LUM), and fibromodulin (FMOD) are members of the small leucine-rich proteoglycan (SLRP) family. DCN is mainly synthesized and secreted by fibroblasts, endothelial cells under stress, and smooth muscle cells [114,115]. It interacts with collagen to regulate collagen fiber formation. BGN connects type II and type VI collagen, organizing VI collagen fibers into hexagonal lattice structures using GAG chains [116,117,118,119]. Moreover, its structural alterations can impact the thickness and morphology of collagen fibers. FMOD, derived from type II SLRPs, consists of a core protein and glycosaminoglycans. It binds to type I and type II collagen, controlling collagen diameter and inter-fiber gaps, thereby influencing and regulating collagen fiber formation, altering the surface characteristics of collagen fibers. Animal experiments involving FMOD knockout found that all collagen fibers showed disorganized arrangement, abnormal morphology, and irregular and rough contours when observed in cross-sections [117]. Overall, these collagens and proteoglycans have a close influence on the generation and arrangement of collagen in the extracellular matrix, and may be the molecular basis for the existence of special collagen structures in interstitial channels.

Furthermore, we found an interesting aspect: essentially, all 72 differential proteins observed in this experiment are associated with gastrointestinal diseases, with some proteins linked to muscular atrophy disorders. In TCM theory, the stomach is the visceral organ connected by this meridian pathway, and muscular atrophy is a condition treatable through acupuncture along this meridian. This seems to suggest that the differential protein expression along this pathway is pathologically related to the stomach, offering some molecular evidence for the meridian–visceral correlation.

Admittedly, this experiment has limitations as it only observed differences in the protein profile under physiological conditions. Further research is needed to investigate protein changes in pathological and acupuncture states. Moreover, there is limited research on proteins in pigs, and many biological processes and metabolic pathways involving these proteins remain unknown. Antibodies sourced from pigs are also limited, allowing validation for only a select few proteins. These aspects will be improved in subsequent experiments.

## 5. Conclusions

In conclusion, the stomach meridian interstitial channels in mini-pigs show 72 differentially expressed proteins compared to adjacent tissues. The differentially upregulated expression of structural proteins (collagen and proteoglycans) forms the foundation for the unique structures in the tissues of this meridian. Their involvement in amino acid metabolism, adenine nucleotide balance conversion, and the elevated expression of functional proteins involved in calcium ion binding suggest the presence of specific biological functions within this pathway and provides new evidence to explain the biological mechanisms underlying the structural and functional specificity of meridians.

## Data Availability

The data presented in this study are available in this article (and Supplementary Materials).

## Supplementary Materials

The following supporting information can be downloaded at https://www.mdpi.com/article/10.3390/biom15060804/s1, Figure S1: Images showing peak areas of all quantified proteins in ST and CT samples before (A) and after (B) normalization; Table S1: Summary of Experimental Controls; Table S2: Predicted structures of all proteins identified.
