# Supplementary material for: Proteomic Study Between Interstitial Channels Along Meridians and Adjacent Areas in Mini-Pigs
Source: Biomolecules. 2025 Jun 1;15(6):804. doi: 10.3390/biom15060804 (PMC12190493; doi:10.3390/biom15060804)

Figure. S1: Images showing peak areas of all quantified proteins in ST and CT samples before (A) and after (B) normalization

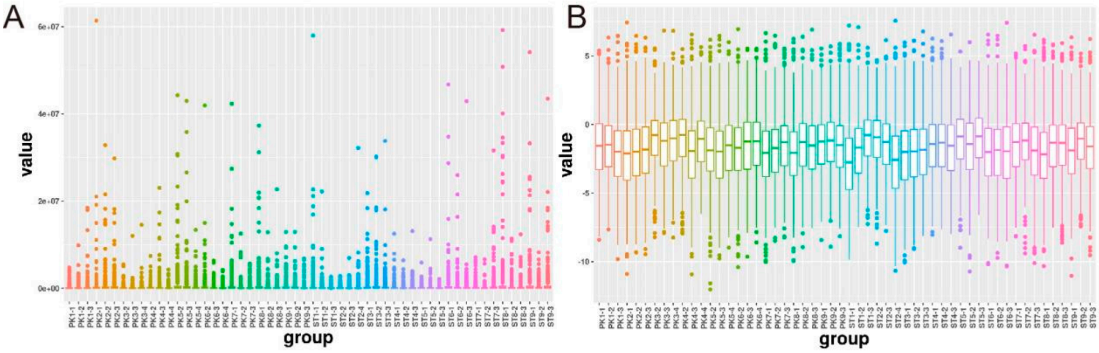

Table S1: Summary of Experimental Controls

Control Type	Purpose	Description	Outcome Validation
**Anatomical (CT)**	Regional specificity control	Non-meridian tissues adjacent to ST	No dye migration; distinct proteomic profile
**Technical (Triplicates)**	Ensure reproducibility	Triplicate SWATH-MS runs per sample	CV < 15% for all quantified proteins

Table S2: Predicted structures of all proteins identified

ID	Structure
A0A286ZVG7	No structure information available for A0A286ZVG7
Q1KYT0	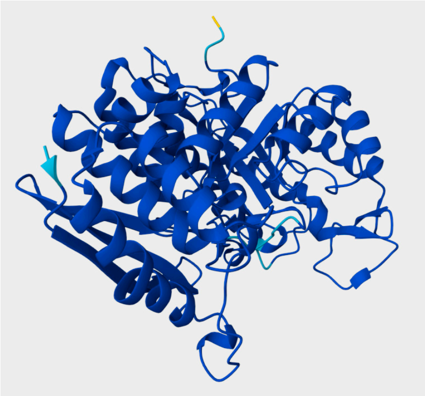

A0A286ZYX8

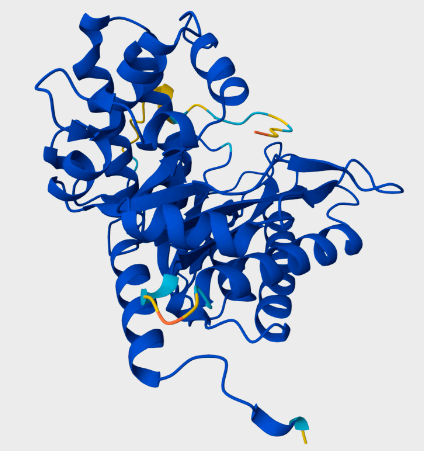

K7GT00 No structure information available for K7GT00

F1SLA6 No structure information available for A0A8D0PY93

Q45EW9

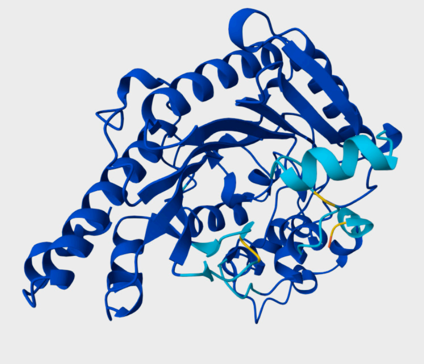

G0Z3A1

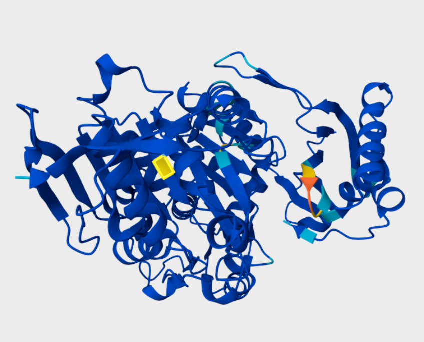

P02543

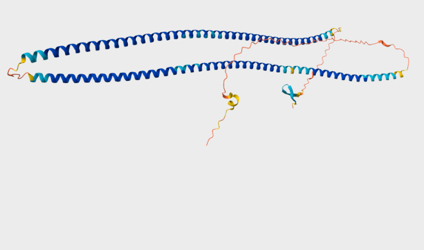

I3LS72

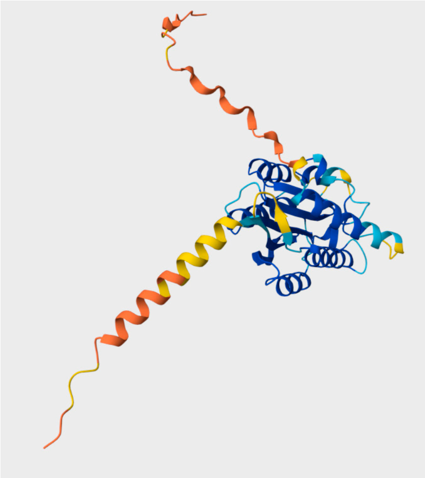

Q5EFJ2

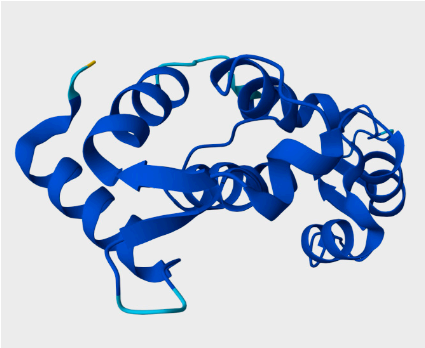

A0A287AE06

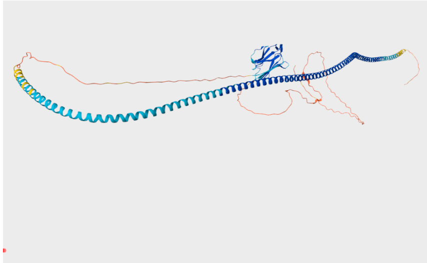

A0A480S5D6	No structure information available for A0A480S5D6
F1RQI0	No structure information available for F1RQI0
A0A287A0A6	No structure information available for A0A287A0A6
NP_998993	No structure information available for NP_998993
A0A4X1TS95	No structure information available for A0A4X1TS95

F1S0V3

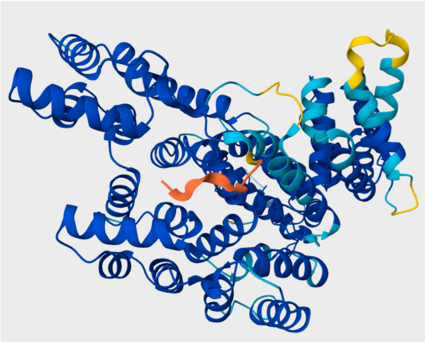

A0A1B2TT55

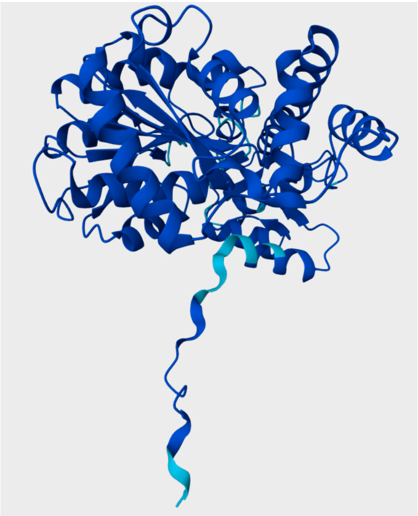

A0A4X1WCQ0

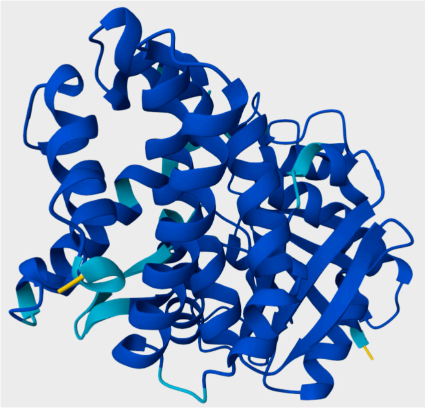

Q9XSH4

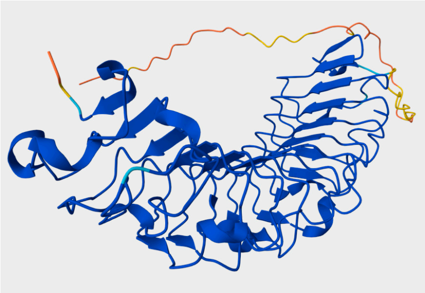

Q8MHY0

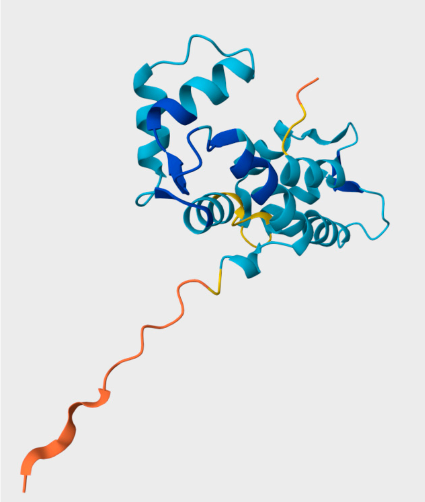

Q2HYU1

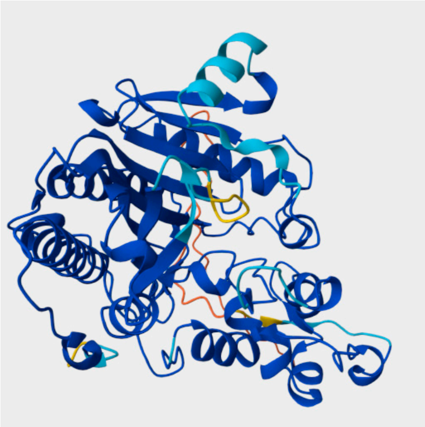

F1S7K4

No structure information available for F1S7K4

A0A286ZYE6

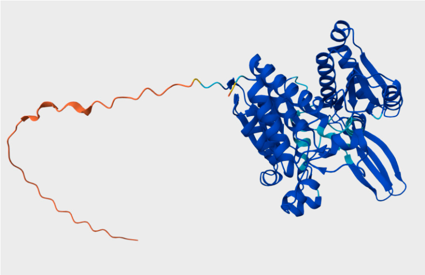

F1SFI5

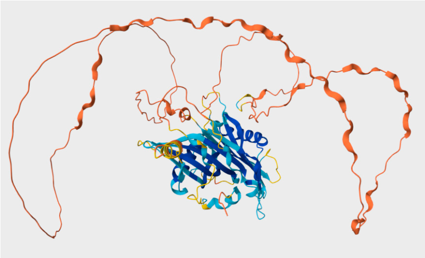

A0A4X1V6K8

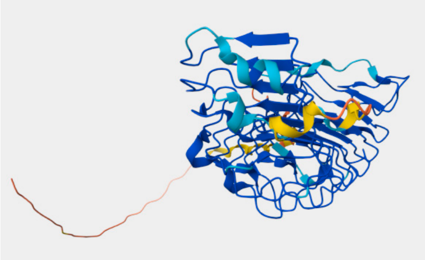

P00571

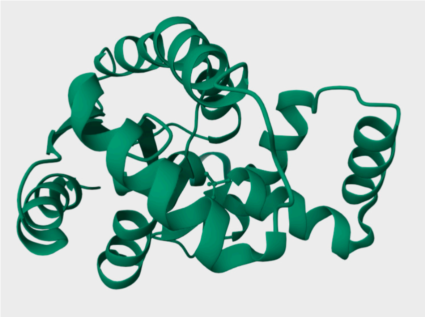

F1S4J7

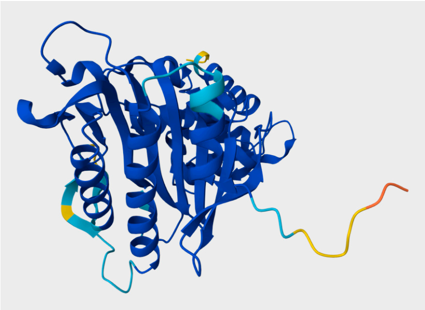

A0A5K1UKC5

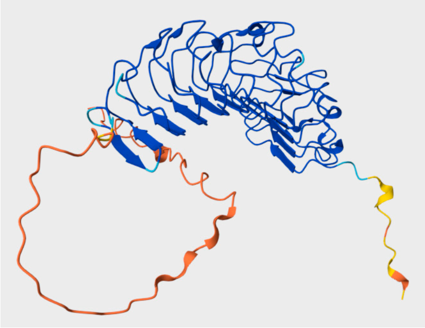

Q9TV63

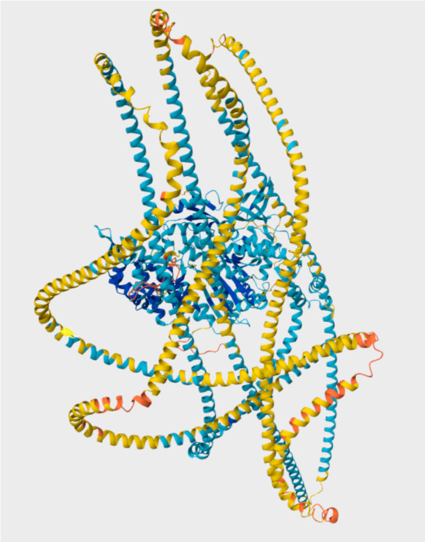

A0A5G2QUU1

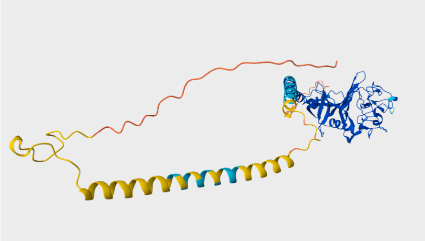

P50447

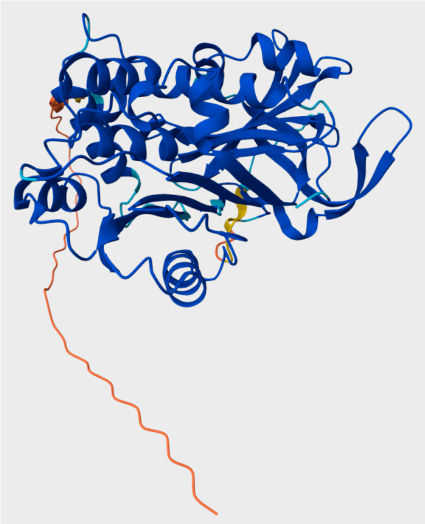

F1SM01

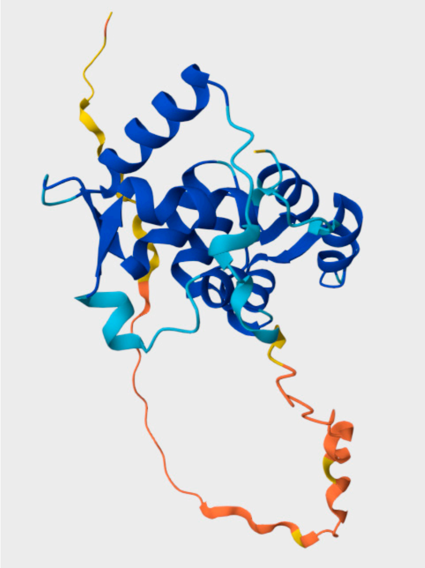

A8U4R4

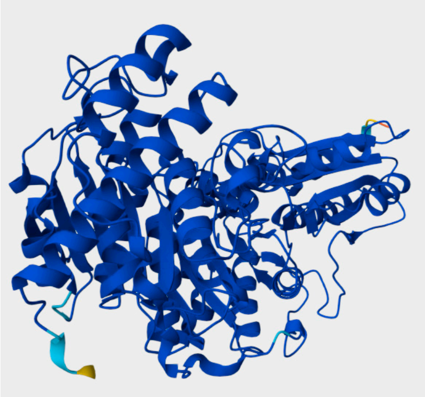

NP_001230248 No structure information available for NP_001230248

Q9TSX9

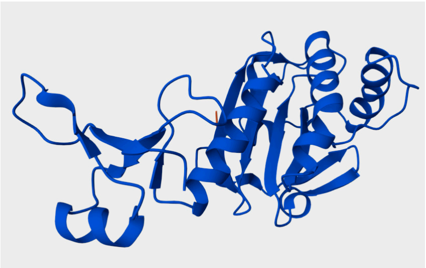

A0A4X1V4X8

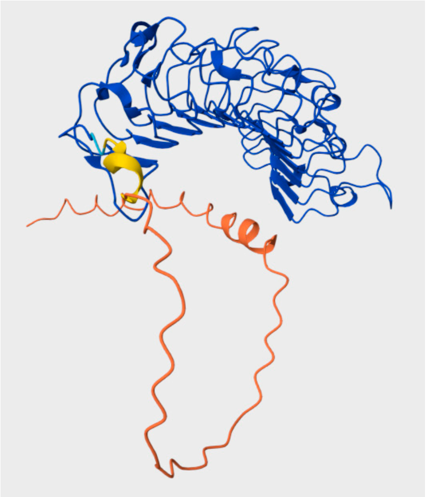

E0X6R2

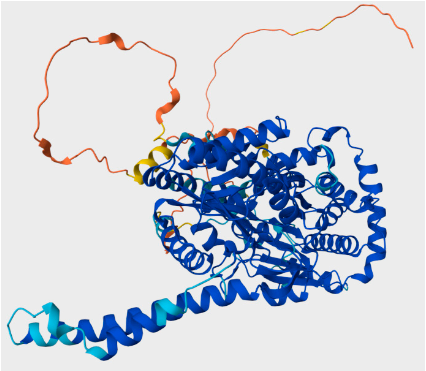

Q56P24

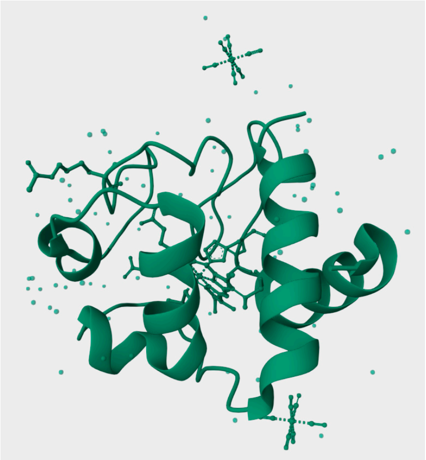

A0A4X1VS41

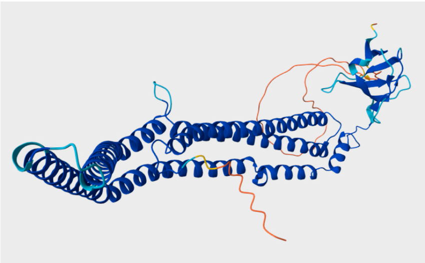

Q5S1U1

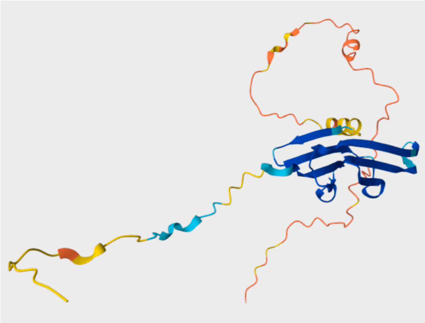

A0PFK6

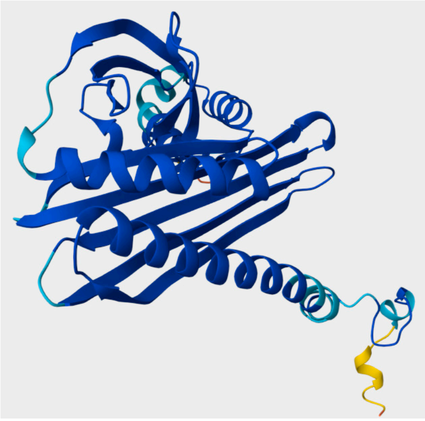

A0A286ZXM0 No structure information available for A0A286ZXM0

I3LDS3

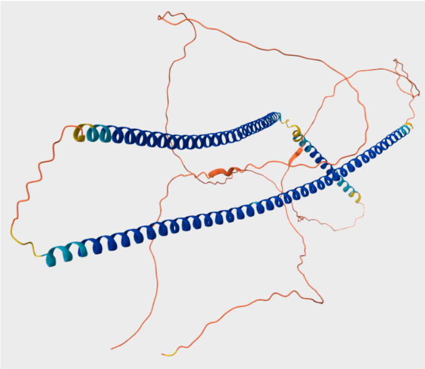

P60662

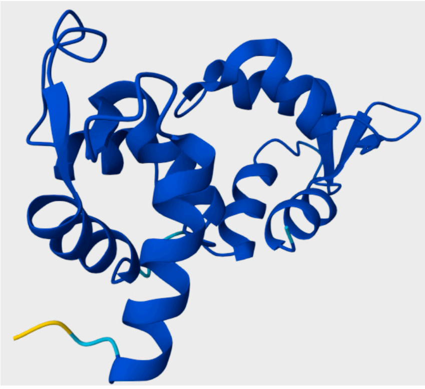

A0A480TTS7

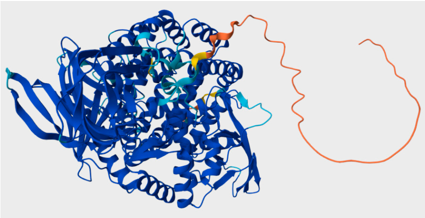

A0A480YSS4

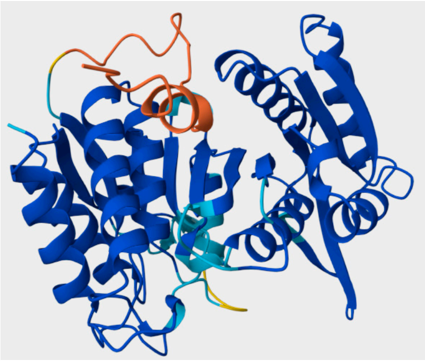

Q6IM67

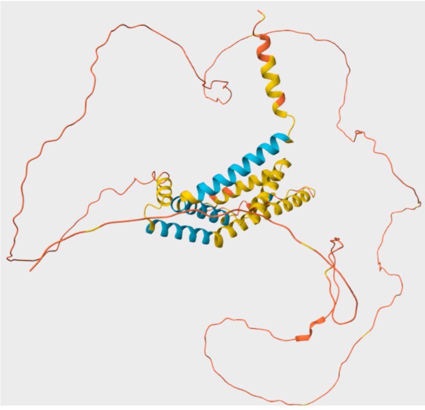

A0A481CJY0

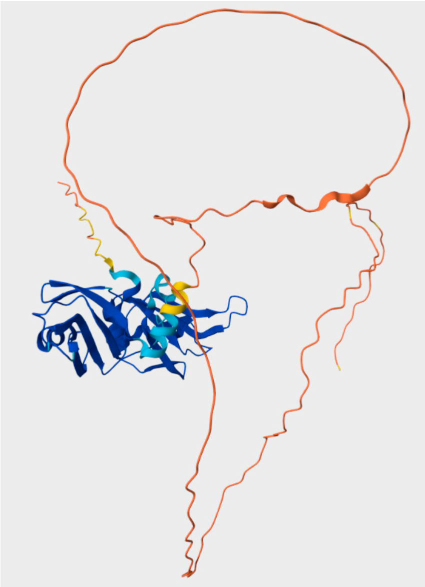

A0A4X1VGR1

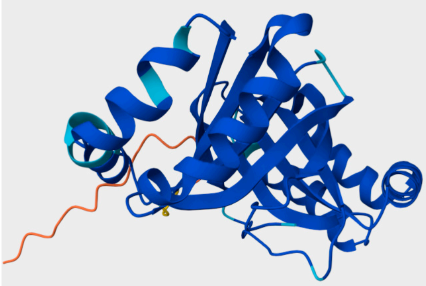

A0A286ZXA4

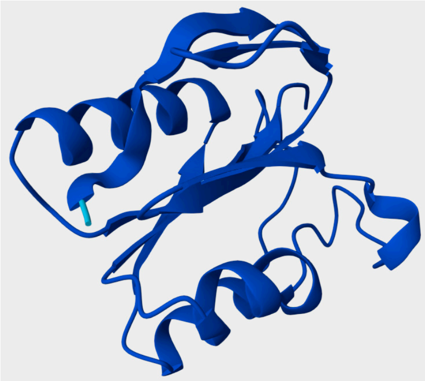

A0A4X1SKD0

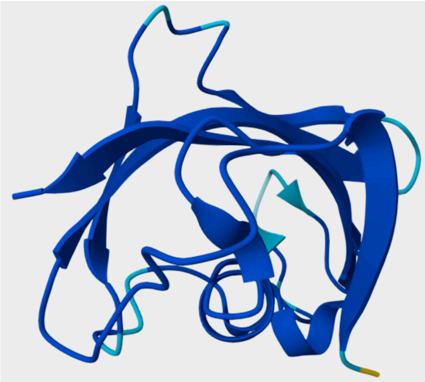

Q9GMB0

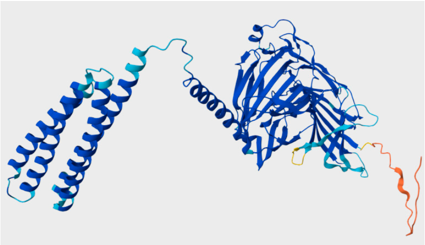

A0A4X1VWC1

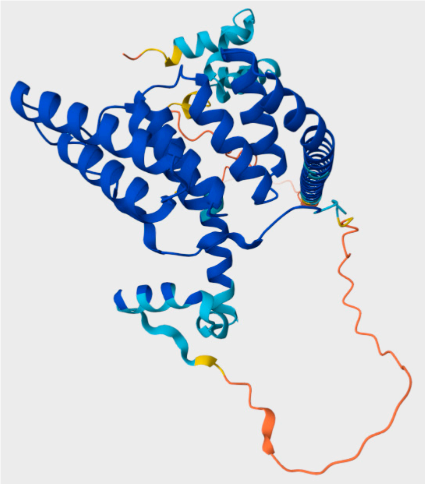

P12309

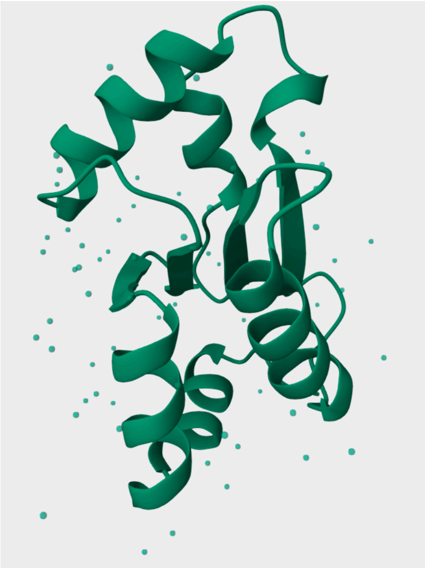

Q007T6

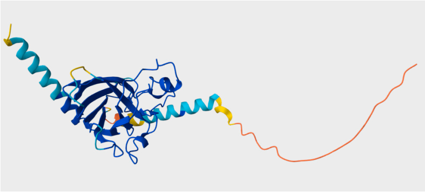

I3LEE6

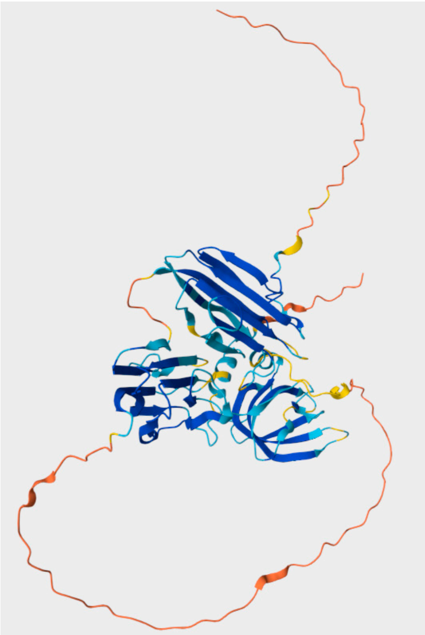

F1STN0

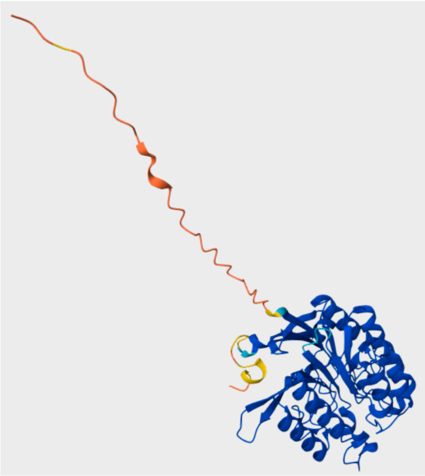

A0A4X1V845

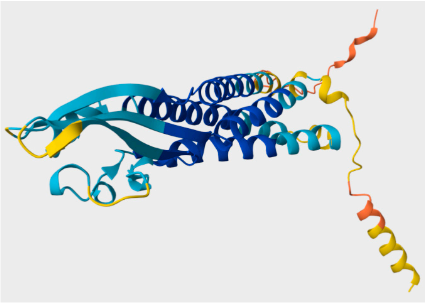

Q6QA25

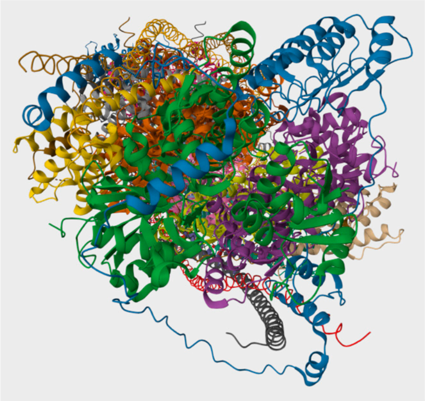

XP_020955779

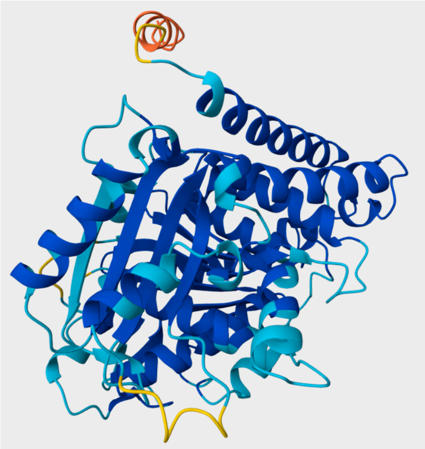

A0A480KM31

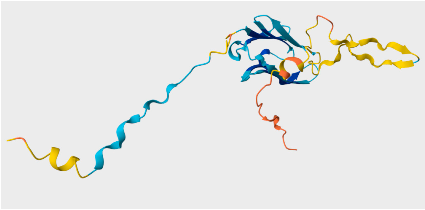

F1SD45

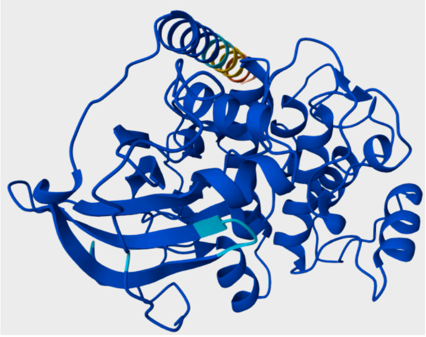

F1RWH7

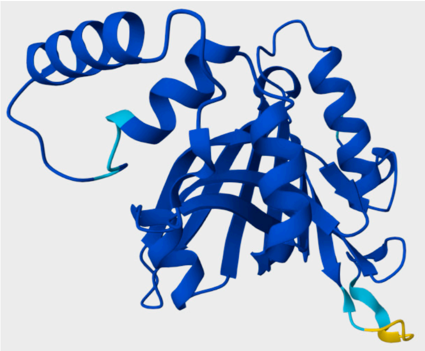

A0A4X1VRG3

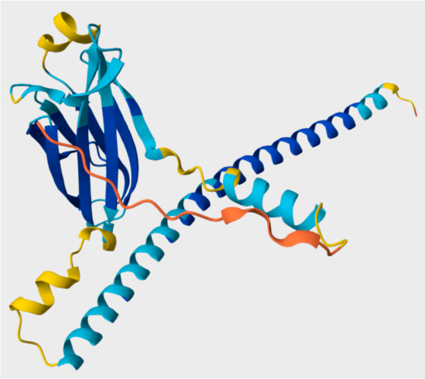

K7GQL2

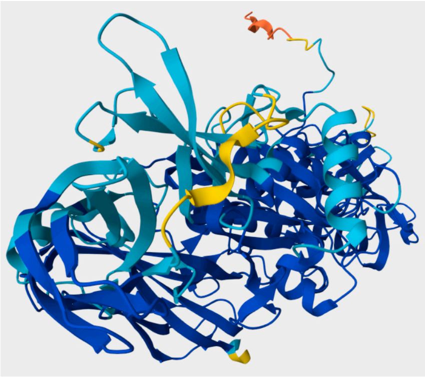

P79381

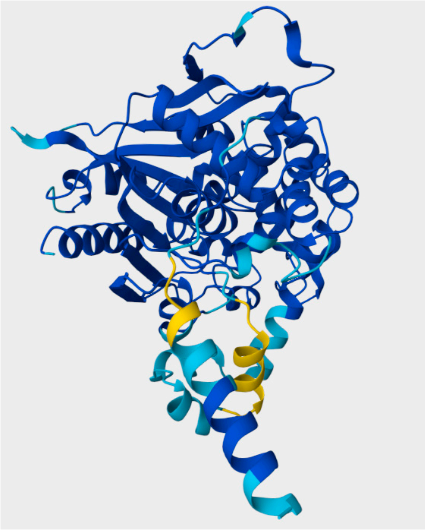

A0A286ZMK8 No structure information available for A0A286ZMK8

Q31072

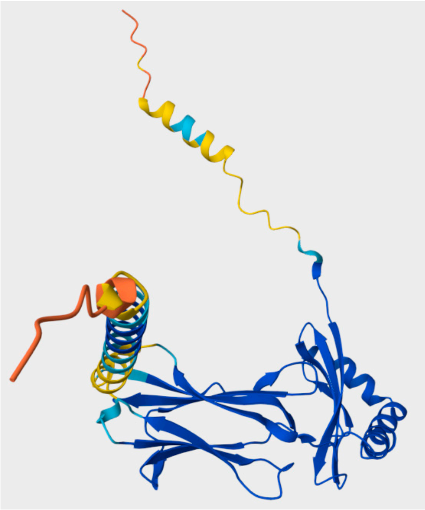

Q8WNR3

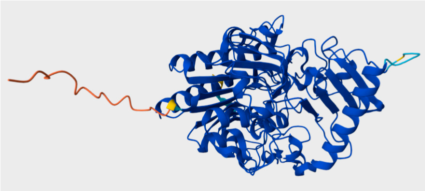

F1S3D5

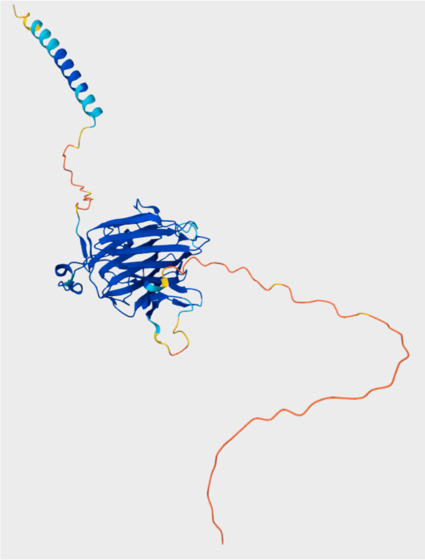

A0A480TGV2

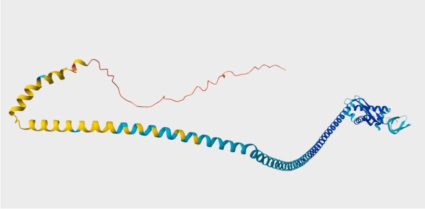

Supplement: Supplementary file 1 [file biomolecules-15-00804-s001.zip › biomolecules-3565363-supplementary.pdf]
